# Four chemometric models enhanced by Latin hypercube sampling design for quantification of anti-COVID drugs: sustainability profiling through multiple greenness, carbon footprint, blueness, and whiteness metrics

**DOI:** 10.1186/s13065-024-01158-7

**Published:** 2024-03-18

**Authors:** Noha S. Katamesh, Ahmed Emad F. Abbas, Shimaa A. Mahmoud

**Affiliations:** 1 Faculty of Pharmacy (Girls), Pharmaceutical Analytical Chemistry Department, Al-Azhar University, Nasr City, Cairo, 11751 Egypt; 2 Faculty of Pharmacy, Analytical Chemistry Department, October 6 University, 6 October City, Giza, 12585 Egypt

**Keywords:** Montelukast sodium, Levocetirizine dihydrochloride, Chemometrics, Latin hypercube design, Greenness evaluation, Whiteness assessment, Blueness evaluation

## Abstract

Montelukast sodium (MLK) and Levocetirizine dihydrochloride (LCZ) are widely prescribed medications with promising therapeutic potential against COVID-19. However, existing analytical methods for their quantification are unsustainable, relying on toxic solvents and expensive instrumentation. Herein, we pioneer a green, cost-effective chemometrics approach for MLK and LCZ analysis using UV spectroscopy and intelligent multivariate calibration. Following a multilevel multifactor experimental design, UV spectral data was acquired for 25 synthetic mixtures and modeled via classical least squares (CLS), principal component regression (PCR), partial least squares (PLS), and genetic algorithm-PLS (GA-PLS) techniques. Latin hypercube sampling (LHS) strategically constructed an optimal validation set of 13 mixtures for unbiased predictive performance assessment. Following optimization of the models regarding latent variables (LVs) and wavelength region, the optimum root mean square error of cross-validation (RMSECV) was attained at 2 LVs for the 210–400 nm spectral range (191 data points). The GA-PLS model demonstrated superb accuracy, with recovery percentages (R%) from 98 to 102% for both analytes, and root mean square error of calibration (RMSEC) and prediction (RMSEP) of (0.0943, 0.1872) and (0.1926, 0.1779) for MLK and LCZ, respectively, as well bias-corrected mean square error of prediction (BCMSEP) of -0.0029 and 0.0176, relative root mean square error of prediction (RRMSEP) reaching 0.7516 and 0.6585, and limits of detection (LOD) reaching 0.0813 and 0.2273 for MLK and LCZ respectively. Practical pharmaceutical sample analysis was successfully confirmed via standard additions. We further conducted pioneering multidimensional sustainability evaluations using state-of-the-art greenness, blueness, and whiteness tools. The method demonstrated favorable environmental metrics across all assessment tools. The obtained Green National Environmental Method Index (NEMI), and Complementary Green Analytical Procedure Index (ComplexGAPI) quadrants affirmed green analytical principles. Additionally, the method had a high Analytical Greenness Metric (AGREE) score (0.90) and a low carbon footprint (0.021), indicating environmental friendliness. We also applied blueness and whiteness assessments using the high Blue Applicability Grade Index (BAGI) and Red–Green–Blue 12 (RGB 12) algorithms. The high BAGI (90) and RGB 12 (90.8) scores confirmed the method's strong applicability, cost-effectiveness, and sustainability. This work puts forward an optimal, economically viable green chemistry paradigm for pharmaceutical quality control aligned with sustainable development goals.

**Supplementary Information:**

The online version contains supplementary material available at 10.1186/s13065-024-01158-7.

## Introduction

The analytical chemistry field faces pressing challenges regarding the environmental sustainability of conventional pharmaceutical analysis techniques [1, 2]. Many established methods diverge from sustainable development objectives, relying extensively on toxic reagents, non-renewable resources, and equipment-intensive procedures that impose major ecological hazards and material waste burdens [3–6]. Chromatographic techniques epitomize these deficiencies, necessitating copious organic solvents like acetonitrile and methanol with immense emissions from production and transport. Additionally, the intricate instrumentation and infrastructure mandate immense energy and financial expenditures, rendering chromatographic platforms unattainable for under-resourced laboratories and propagating global analytical disparities [7].

This status quo demands urgent solutions through pioneering analytical techniques embracing green analytical chemistry (GAC) and white analytical chemistry (WAC) philosophies [8, 9]. The widely embraced 12 GAC Principles delineate pragmatic guidelines like minimizing material inputs, preventing waste, utilizing renewable materials, and enabling real-time analysis with minimal pre-treatment. Complementarily, WAC focuses on economical, adaptable, easily disseminated methods to democratize analytical science and overcome global analytical inequities. By emphasizing decreased hazards, instrumentation burdens, and costs, WAC facilitates universal adoption. Within this context, UV–visible spectrophotometry (UV–vis) has emerged as an excellent technique for furthering GAC and WAC objectives. Requiring only inexpensive reagents and basic equipment with minimal waste, UV–vis aligns well with GAC aspirations and WAC accessibility goals [10, 11].

However, direct pharmaceutical quantification often fails due to substantial spectral overlaps obscuring individual component signals like active pharmaceutical ingredients. This motivated the integration of UV–vis with chemometrics, establishing a synergistic combination extracting meaningful chemical information from complex backgrounds, quintessential in analytical chemistry [12]. Thereby transforming UV spectrophotometry into a value-added green analytical tool suitable for economical, routine pharmaceutical quality control, as evidenced in recent literature [13–15].

Nevertheless, prevailing chemometrics studies predominantly use random data splitting for training and validation subsets [14, 15]. While simple to implement, random partitioning risks insufficiently representing the full modeled chemical space, frequently causing biased accuracy estimates conflicting with sustainability objectives like reliability and resource efficiency. To address this significant limitation, we strategically leverage the Latin Hypercube sampling (LHS) design, systematically dividing each modeled variable into equal probability strata and sampling each to ensure excellent coverage and balance when constructing validation sets [13]. Thereby enabling robust, unbiased assessment of predictive capabilities on new samples. Compared to excessive random sampling, LHS provides equivalent predictive performance testing with substantially fewer validation experiments. This enhances greenness through more efficient resource utilization while preventing misleading model accuracy estimates that could compromise pharmaceutical quality control adoption. Thus, LHS holds immense untapped potential for developing reliable, sample-efficient chemometrics methodologies aligning with sustainable growth.

Propelled by the potential of LHS and multivariate UV spectrophotometry for sustainable pharmaceutical analysis, this work pioneers LHS for constructing validation sets when quantifying active pharmaceutical ingredients via chemometrics. We coupled LHS with genetic algorithm optimization of the most information-rich spectral subsets to maximize predictive performance. To demonstrate capabilities, two dissimilar extensively used pharmaceuticals were targeted—montelukast sodium (MLK) and levocetirizine dihydrochloride (LCZ). In 2020, MLK was the 14th most prescribed drug in the United States with over 31 million prescriptions, owing to its use as a leading repurposed COVID-19 therapeutic [16, 17]. Meanwhile, LCZ attained over 3 million 2020 prescriptions due to its synergistic administration with MLK as an anti-COVID agent [18]. Nevertheless, existing literature lacks green analytical methods for quantifying this vital MLK-LCZ combination to support sustainable pharmaceutical manufacturing and quality control.

**Fig. 1:**
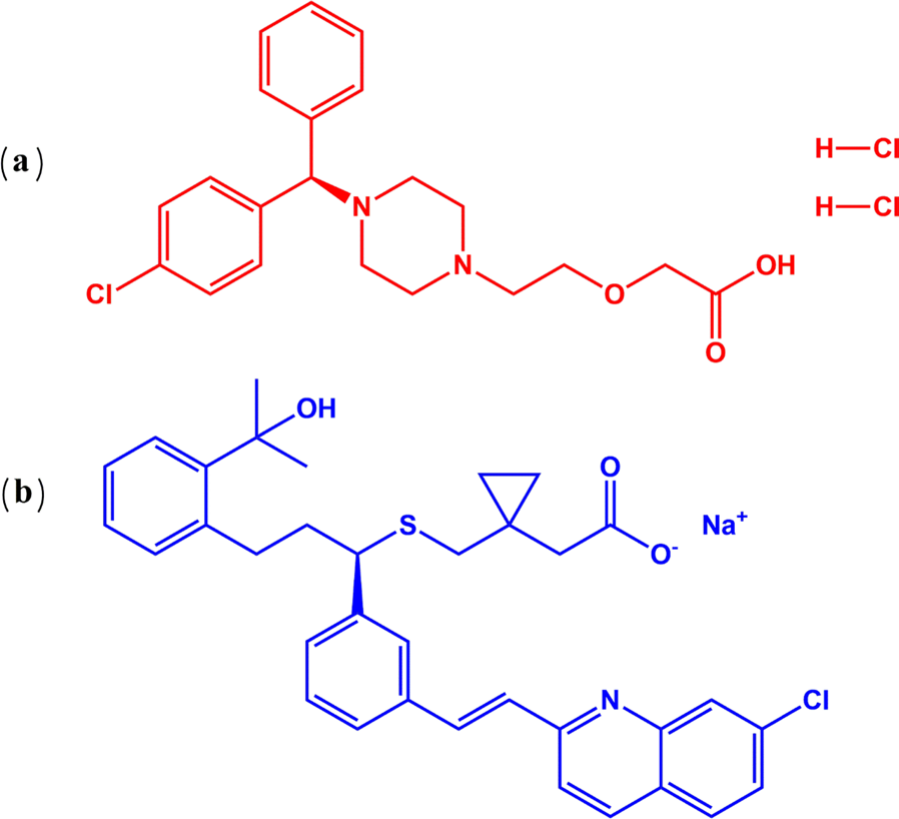
Chemical structure of **a** LCZ, and **b** MLK

MLK (Fig. 1) is 2-[1-[[(1R)-1-[3-[2-(7-chloroquinolin-2-yl)ethenyl]phenyl]-3-[hydroxypropan-2yl)phenyl]propyl]sulfanylmethyl]cyclopropyl] acetic acid sodium salt. LCZ (Fig. 1) is (2-(4-((R)-(4-chlorophenyl)phenylmethyl)-1-piperazinyl)ethoxy) acetic acid dihydrochloride. While several techniques have quantified both drugs in combination, including spectroscopic [19–21], HPLC methods [22–26], two TLC methods [26, 27], and one UPLC method [28], these remain expensive, complex, utilize non-green solvents, and are unsuited for routine analysis. This necessitates alternative techniques harmonizing with GAC and WAC principles to balance efficacy and eco-friendliness. To our knowledge, no chemometric methods incorporating green–blue-white analysis have been reported to simultaneously determine MLK and LCZ.

This work contributes to addressing these gaps by combining UV–vis spectroscopy's green advantages with multivariate calibration and strategically constructed LHS validation sets to optimize accuracy across the modeled pharmaceutical space. We further incorporated cutting-edge sustainability metrics spanning greenness, blueness, and whiteness to enable multifaceted comparisons with existing methods. The overarching objectives are: (1) construct enhanced multivariate models via LHS and genetic algorithms for selective MLK and LCZ analysis without chromatographic separation; (2) demonstrate chemometrics as a valuable green analytical alternative for economical, routine quality control; (3) comprehensively evaluate sustainability using tools like NEMI, ComplexGAPI, AGREE and carbon footprint to affirm favorable greenness; (4) spearhead “blueness” and “whiteness” assessments via pioneering BAGI and RGB12 metrics to validate analytical performance, cost-effectiveness, and potential for widespread pharmaceutical laboratory implementation.

## Experimental

### Instrumentation and software

A Shimadzu UV-1800 double-beam spectrophotometer equipped with 1 cm quartz cells was utilized for spectral data acquisition. The UV-Probe software version 2.42 controlled the measurements, obtained at a 1.0 nm slit width using a fast single scan mode with 0.1 nm sampling interval. Additional instrumentation included an ultrasonic bath (Julabo Labortechnik, Germany) for extraction and a Shimadzu analytical balance (AGE-220) for weighing. Data processing and chemometrics analysis were performed with Matlab R2013a with PLS Toolbox v2.0. Excel-enabled ANOVA statistical analysis.

### Reagents and materials

Ultrapure water (Milli-Q, Millipore) was used throughout the procedures. All chemicals were of analytical grade. Reference standard compounds of montelukast sodium (MLK; batch no. MK-0180513) and levocetirizine dihydrochloride (LCZ; batch no. LCZ-1304009) were obtained from EGY Pharm with certified purity of 99.30% and 99.78%, respectively. Montair-LC® tablets (batch no. D3184-8) produced by Cipla (India) and labeled to contain 10 mg MLK and 5 mg LCZ per tablet were procured from a local pharmacy for analysis.

### Standard solutions

Individual stock solutions of 100 μg/mL MLK and LCZ were prepared by accurately weighing 10 mg reference standard into a 50 mL volumetric flask, dissolving in water, and making up to final volume. Stock solutions demonstrated stability for one month under refrigeration (4 °C). Working standard solutions were freshly prepared daily by appropriate dilution of stock solutions with ultrapure water to yield the desired concentration range.

### Spectral characteristics and linearity

**Fig. 2:**
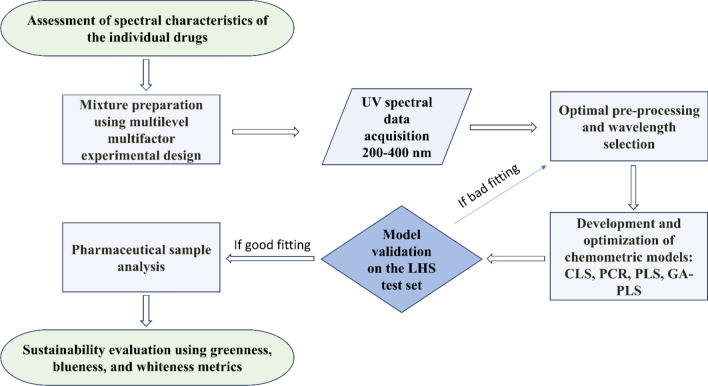
Flowchart visualizes the key steps in the methodology

**Fig. 3:**
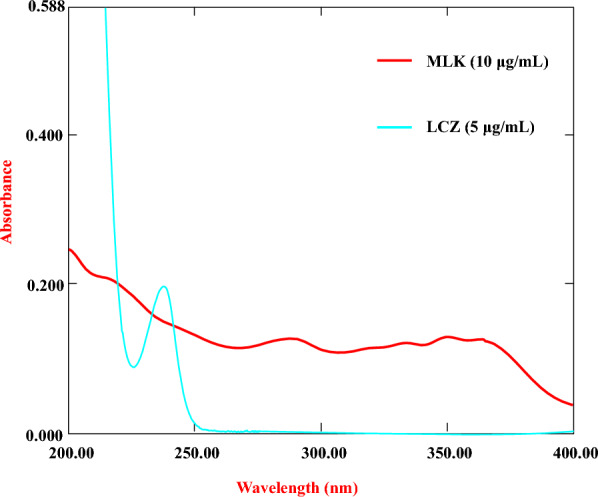
The zero-order absorption spectrum of LCZ, and MLK

Assessment of spectral characteristics is an important initial phase to understand the UV absorption profiles and spectral overlap between the analytes, as shown in (Fig. 2). To assess spectral characteristics, the UV absorption spectra of MLK and LCZ were recorded individually over the wavelength range of 200–400 nm. The scans were performed for standard solutions of each component prepared at concentrations of 10 μg/ml for MLK and 5 μg/ml for LCZ, as illustrated in (Fig. 3). These concentrations were selected within the typical linear dynamic ranges and pharmaceutical ratio. To assess the linearity of the method, UV spectra of mixtures containing MLK and LCZ were obtained at intervals of 1 nm from 200 to 400 nm, with concentration ranges of 10–30 μg/ml for MLK and LCZ. These ranges were selected based on the typical concentrations expected in pharmaceutical samples and to cover the linear dynamic range of the instrument.

### Experimental design

A systematic experimental framework is imperative for acquiring representative, information-rich spectral data. A multilevel multifactor calibration set of 25 mixtures was constructed based on the design of Brereton et al. [29]. This generated a calibration set of 25 mixtures containing varying proportions of 10–30 μg/ml for MLK and LCZ. The validation set was prepared using LHS, which ensures representative sampling of the concentration space for reliable model validation [30]. The concentration range was divided into 13 equal probability strata from which 13 distinct mixtures were sampled for the validation set. Both designs align with GAC and WAC as they have many advantages, including simplicity, sensitivity, selectivity, user-friendliness, cost-effectiveness, minimal solvent usage, time efficiency, and eco-friendliness.

Mixtures were prepared in 25 ml volumetric flasks using micropipettes and ultrapure water as solvent. Absorption spectra from 200–400 nm were acquired against a water blank with 1 cm quartz cuvettes. Spectral regions below 210 nm and above 400 nm were discarded due to noise and lack of signals, respectively, yielding the working spectral data matrix of 210–400 nm at 1 nm resolution (191 data points). This spectral data was utilized for the development and validation of the chemometrics models.

### Models building and optimization

Four chemometric regression approaches were implemented for the model building including classical least squares (CLS), principal component regression (PCR), partial least squares (PLS), and PLS coupled with genetic algorithm wavelength selection (GA-PLS). Rigorous calibration model optimization was performed using the 25-mixture design calibration set to avoid overfitting and derive maximally robust models. For CLS, the regression was calculated independently at each measured wavelength without compression into latent variables (LVs). However, a moving window wavelength selection approach was utilized to optimize model performance, where window widths from 5 to 30 nm were evaluated through cross-validation to determine the optimal spectral smoothing level, balancing noise reduction with retention of quantitative information. For PCR and PLS, the optimal number of LVs was systematically varied from 1 to 10 and identified through Venetian blinds cross-validation, monitoring root mean squared error of cross-validation (RMSECV). The selected LVs number balanced model fit and complexity. for GA-PLS, GA variables were optimized to extract the most information-rich and noise-minimized portion of the spectra for importation into a refined PLS model, balancing predictive ability, reliability, and generalization capability based on the calibration set After optimization, the models were applied to determine MLK and LCZ in the external validation set mixtures to evaluate predictive performance.

### Figures of merit

Multiple essential metrics were computed to thoroughly evaluate the predictive capacity, precision, sensitivity, accuracy, and robustness of the fully optimized chemometrics models [31]. The root mean square error of calibration (RMSEC) and RMSECV were determined using the calibration set spectra as measures of model fitting. Additionally, the standard error of calibration (SEC) and explained variance (R^2^) quantified goodness of fit.

Predictive ability was evaluated through the cross-validated predictive ability (Q^2^) metric computed by systematic cross-validation on the calibration set. The predictive accuracy on new samples was quantified through the relative root mean square error of prediction (RRMSEP) and root mean square error of prediction (RMSEP) metrics on the external validation set. Furthermore, the bias-corrected mean square error of prediction (BCMSEP) gauged the precision and variance of predictions.

The equations used to determine R^2^, Q^2^, RMSECV, RMSEP, and RMSEC are as follows [31]:

$$Residual\,Sum\,of\,Squares (RSS) = \Sigma {({ y}_{observed}-{y}_{predicted} )}^{2}$$

$$Total\,Sum\,of\,Squares (SSX)= \Sigma {({ y}_{observed}-{y}_{mean} )}^{2}$$

$${R}^{2}= 1 - (RSS / SSX)$$

$$Predictive\,Residual\,Sum\,of\,Squares (PRESS)= \Sigma {({ y}_{observed}- {y}_{{predicted}_{CV}} )}^{2}$$

$${Q}^{2}= 1 - (PRESS / SSX)$$

$$RMSE=\sqrt{\frac{{\sum }_{i=1}^{n}{(yi - \widehat{y}i)}^{2}}{n}}$$

The remaining figures of merit were calculated using the following equations:

$$Bias=\frac{{\sum }_{i=1}^{n}(yi - \widehat{y}i)}{n}$$

$$SEC=\sqrt{\frac{{\sum }_{i=1}^{n}{(yi - \widehat{y}i -bias)}^{2}}{n-1}}$$

$$RRMSEP\%=\frac{\frac{1}{n}\sqrt{{\sum }_{i=1}^{n}{(yi - \widehat{y}i )}^{2}}}{\overline{y}i }\times 100$$

$$BCMSEP=\frac{{\sum }_{i=1}^{n}{(yi - \widehat{y}i)}^{2}}{n} - {(bias)}^{2}$$

The value $\widehat{y}i$ represents the outcome acquired during the process of calibration (in the case of RMSEC), validation (in the case of RMSEP), and cross-validation (in the case of RMSECV). The variable $yi$ denotes the experimental result for the sample $i$, while n represents the total number of samples.

To gauge accuracy, triplicate measurements were conducted at three different concentration levels within the linear range for each analyte (15, 20, and 25 µg/mL), followed by the calculation of percent recoveries (%R). Repeatability (intra-day) and intermediate (inter-day) precision were assessed by analyzing triplicate samples on the same day and on three separate days, respectively, at concentrations of 15, 20, and 25 µg/mL for MLK and LCZ, with percent relative standard deviation (%RSD) used to quantify precision. Robustness was investigated by making slight modifications to experimental variables, such as wavelength interval (0.9 nm instead of 1 nm), spectral bandwidth (0.8 nm instead of 1 nm slit width), and scan speed (medium instead of fast scan). The capability to withstand these alterations demonstrated method robustness. The limits of detection (LOD) and quantification (LOQ) were calculated based on the standard error of the regression curve created between experimentally measured concentrations (y-axis) and concentrations predicted by the models (x-axis), using the IUPAC-recommended equations [11]:

$$ {\text{LOD }} = { 3}.{3 }\sigma /{\text{S}} $$

$$ {\text{LOQ }} = { 1}0 \, \sigma /{\text{S}} $$

where σ is the standard error of the prediction-vs-actual regression quantifying variability in predictions, and S is the corresponding slope indicating sensitivity to concentration changes. This extensive analytical validation provided insights into the predictive capabilities, accuracy, precision, sensitivity, and robustness of the developed models on pharmaceutical mixture analysis.

### Analysis of pharmaceutical dosage forms

Five Montair-LC® tablets (label claim: 10 mg MLK and 5 LCZ per tablet) were crushed together to a fine powder. A precisely measured amount of powder, equal to the weight of one tablet, was placed into a volumetric flask with a capacity of 100 mL. Approximately 50 mL of water was added and sonicated for 15 min to extract the drugs into the solution. The volume was made up of water, mixed well, passed through a membrane filter with a pore size of 0.45 μm and appropriately diluted with water to obtain concentrations of (100 μg mL^−1^ MLK, and 50 μg mL^−1^ LCZ). Suitable aliquots were transferred from the clear filtrate into 10 mL volumetric flasks and diluted with water. The absorption spectra of these diluted sample solutions were recorded in the range of 200–400 nm against a water blank. The spectra were analyzed employing the developed chemometrics models to determine the concentrations of MLK and LCZ in the pharmaceutical preparation. The accuracy was evaluated by spiked standard addition at four concentration levels in triplicate. R % and %RSD were calculated.

## Results and discussion

### Spectral data acquisition and chemometrics models

The development of an accurate and reliable analytical method requires systematic optimization of experimental parameters to extract the maximum relevant chemical information. For spectral acquisition, key parameters optimized included wavelength range, sampling interval, scan speed, and slit width. A wavelength range of 200–400 nm was selected by scanning standard solutions of MLK and LCZ to identify regions exhibiting significant absorbance peaks. This encapsulated the full fingerprints of both analytes. The sampling interval was set to 1 nm to provide sufficient resolution to discern subtle spectral features important for quantification. Additionally, a fast scan speed was chosen to enable higher throughput analysis while using a slit width of 1 nm balanced resolution with sufficient light throughput. These acquisition parameters delivered a spectral data matrix with optimal information content and signal-to-noise ratios to support chemometrics modeling. Preprocessing identified the working range of 210–400 nm (191 data points) as optimal by removing uninformative noise at higher and lower wavelengths. The resulting high-quality spectral data matrix was then subjected to iterative chemometrics optimization.

A range of chemometrics techniques including CLS, PCR, PLS, and GA-PLS were implemented. These approaches were chosen based on their demonstrated ability to handle multi-component analysis and extract latent predictive information even with significant collinearity and interactions between constituents [13, 15]. This enabled accurate quantification despite the significant spectral overlaps seen in Fig. 3. Overall, this chemometrics-powered workflow achieved sensitive pharmaceutical determination while aligning with green chemistry aims through efficient use of the information-rich and cost-effective UV–vis fingerprint region.

#### Calibration set design

A key facet of developing an accurate multivariate calibration model is the strategic design of the calibration sample set. A poorly conceptualized calibration set risks insufficient representation of the analytical system's variation, leading to a lack of model robustness. To avoid this, we utilized a multilevel multifactor experimental design proposed by Brereton et al. [29] to systematically construct an optimized 25-sample calibration set with varying proportions of MLK and LCZ at five different concentration levels (− 2, − 1, 0, + 1, + 2), as shown in (Table 1). The concentration ranges for the calibration set were selected based on the typical concentrations expected in pharmaceutical samples and to cover the linear dynamic range of the UV instrument. For the calibration set, concentrations of 10–30 μg/ml for both MLK and LCZ were used. This range spans the linear range of the method and the typical pharmaceutical concentrations of the two analytes. Compared to using excessive calibration samples, this structured approach provides several important advantages. First, it allows the intentional creation of non-correlated concentration profiles between the analytes. Keeping the calibration concentration vectors orthogonal prevents covariation and enables the model to better discern the unique spectral contribution of each analyte. This enhances selectivity and prevents overfitting artifacts that can reduce predictive accuracy for complex samples. Second, the use of multiple concentration levels for both analytes ensures variation across all dimensions of the calibration space. This enhanced representation helps the model effectively identify and weight the spectral regions correlating with analyte concentration. Capturing this multivariate concentration-spectral relationship is key for quantification. Finally, the hierarchical nature of the experimental design minimizes the number of samples required to systematically span the calibration space. By strategically selecting samples, this approach reduces resource usage, chemical waste, instrumental analysis time, and overall experimental cost compared to excessive calibration samples. The improved efficiency makes this an aligned green analytical chemistry practice. Overall, the multilevel multifactor calibration design improved predictive performance and greenness through efficient, structured sampling and modeling of the concentration space for all analytes. This aligns well with the core principles of developing sustainable analytical methods.

**Table 1:** The five-level five-factor experimental design of 25 calibrations mixtures and the Latin Hypercube sampling design 13 validation set mixtures used in the chemometric methods

Mix no.	Calibration set (µg/mL)	
	LCZ	MLK
1	20	20
2	20	10
3	10	10
4	10	30
5	30	15
6	15	30
7	30	20
8	20	15
9	15	15
10	15	25
11	25	30
12	30	25
13	25	20
14	20	30
15	30	30
16	30	10
17	10	25
18	25	10
19	10	20
20	20	25
21	25	25
22	25	15
23	15	10
24	10	15
25	15	20

Mix no.	Validation set (µg/mL)	
	LCZ	MLK
1	19	16
2	22	27
3	13	12
4	22	13
5	18	26
6	11	22
7	16	29
8	17	21
9	27	11
10	27	24
11	13	27
12	29	19
13	23	21

#### Validation set design

**Fig. 4.**
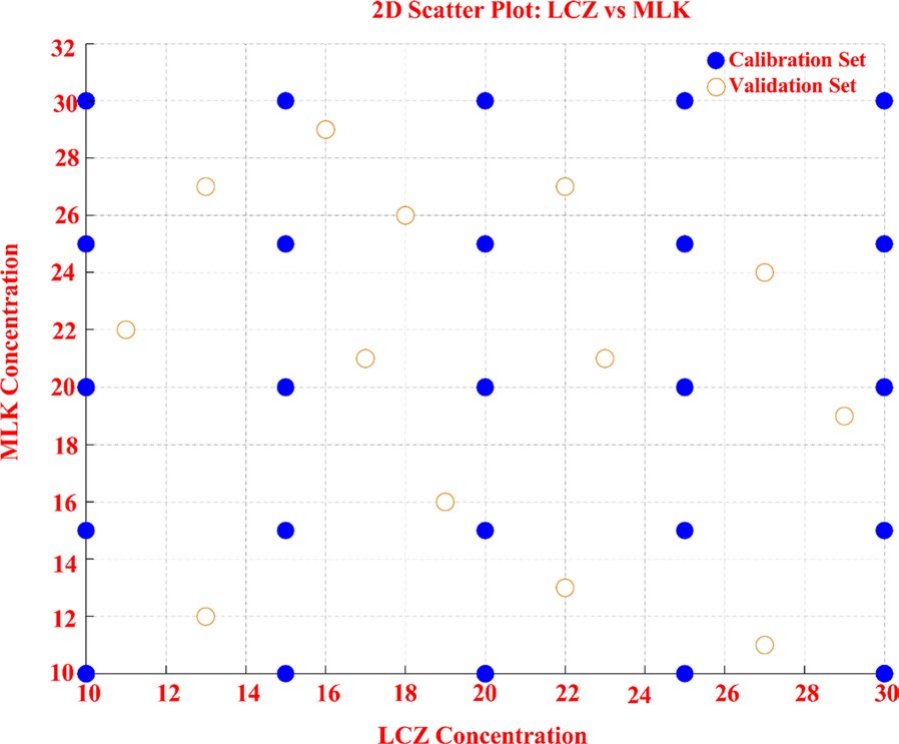
2D scatter plot of the validation set designed by latin hypercube sampling design as optimal-space filling design

Proper design of the validation set was critical for unbiased assessment of the chemometrics models’ predictive accuracy across diverse combinations of analytes. Simple random sampling risks incomplete coverage that produces biased accuracy estimates. To overcome this critical limitation, we systematically designed the validation set using LHS, a statistically efficient space-filling experimental design technique. LHS divides the full concentration range of each modeled component into N equal probability strata, with N chosen to balance model evaluation needs with green analytical principles of efficiency. We determined an optimal validation set size of 13 LHS-selected mixtures based on the number of modeled factors and mixtures in the calibration set, as shown in (Table 1). Using a prime number avoids potential periodic resonances between stratification levels. The 13 strata provide wide coverage while the prime number selection enhances space-filling properties. LHS selects exactly one sample from each of the 13 strata, ensuring even and comprehensive coverage across all dimensions of the modeled pharmaceutical concentration space. This is visualized in the scatter plots of (Fig. 4), showing the 13 LHS validation samples achieving excellent uniform scattering across all analyte ranges without gaps. Compared to simplistic random sampling, LHS provides superior concentration space coverage and representativeness using significantly fewer samples. By improving concentration space sampling efficiency, LHS allows a smaller but more informative and representative validation set. This enhances method greenness by reducing material usage, waste, and cost. Additionally, the reliable predictive performance estimates on this strategically designed LHS test set demonstrate the robustness and generalization capability of the model across varying and diverse pharmaceutical component compositions. This avoids biases or exaggerated accuracy that could occur with insufficient validation set sampling via random selection. Overall, LHS overcomes critical limitations of simplistic approaches, enabling trustworthy, sample-efficient assessment of predictive performance across the modeled pharmaceutical concentration space. This aligns well with core principles of developing sustainable analytical methods.

#### CLS model

The CLS method employs multivariate linear regression based on the Beer-Lambert law, requiring accurate analyte spectral profiles and concentrations for all calibration samples [15]. A key assumption in CLS is that a linear relationship exists between absorbance and component concentration across the modeled spectral region. Initial CLS models built on the 25-mixture calibration set yielded inadequate predictions. However, incorporating an intercept term into the CLS algorithm significantly improved results, yielding excellent recovery percentages of 99.28% and 101.44% for MLK and LCZ, respectively. This demonstrates that an intercept adjustment enabled the CLS method to account for subtle nonlinear behaviors and background effects. While straightforward in principle, CLS has intrinsic limitations when complex nonlinear interactions or unknown constituents are present, constraining predictive accuracy. Its requirement for precise reference spectra for all sample components is often impractical for complex pharmaceutical mixtures. However, CLS provided an accessible starting point for analysis in this work before more advanced chemometric methods demonstrated superior performance.

#### PCR model

**Fig. 5:**
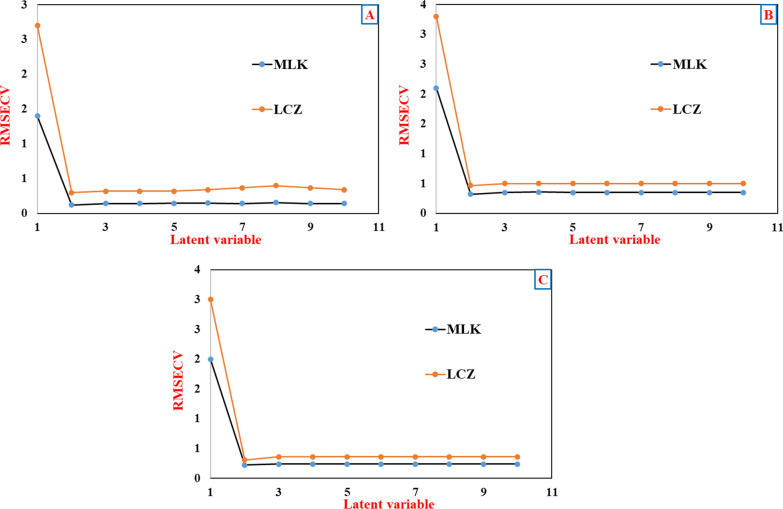
RMSECV plot of the calibration set as a function of the optimum LVs for the **A** PCR, **B** PLS, and **C** GA-PLS models

PCR model combines multivariate regression with principal component analysis (PCA) for predictive modeling [15]. This two-step approach first applies PCA to the spectral data matrix to extract major trends while reducing noise, artifacts, and collinearity. PCA generates new orthogonal explanatory variables called principal components (PCs) that successively capture the major variation within the spectra. Cross-validation was employed to determine the optimal number of PCs to retain for the highest predictive accuracy without overfitting. The 25-mixture calibration set spectra were mean-centered, and PCR models were constructed by systematically excluding one sample during each cross-validation round. Root mean square error of cross-validation (RMSECV) was monitored with increasing numbers of PCs included in the PCR model. Typically, prediction error decreases initially as informative PCs are added then worsens as noise dominates higher-order PCs. The optimal complexity balancing model fit and generalization was two PCs for both pharmaceutical components. This extracted the key spectral variations related to API concentrations while discarding interfereing contributions. The two-PC PCR model yielded RMSECV values of 0.121 and 0.297 for MLK and LCZ respectively, indicating good predictive performance on the calibration set itself, as illustrated in (Fig. 5).

#### PLS model

PLS is a prominent chemometrics technique closely related to PCA and regression. However, PLS has a distinct objective—rather than explaining spectral variation like PCA, PLS aims to maximize the covariance between spectral data (predictor variables) and component concentrations (response variables). This retains LVs directly related to concentration prediction while discarding interfering spectral contributions uncorrelated with analyte levels. PLS builds these LVs sequentially, concentrating information co-varying with concentration into earlier factors. By retaining information-rich regions and discarding uninformative spectral bands, PLS typically achieves improved accuracy compared to PCR [15]. The optimal PLS model complexity was determined through leave-one-out cross-validation on the 25-mixture calibration set. The number of latent variables was systematically increased, monitoring RMSECV to balance model fit against overfitting. Ideally, 2 LVs proved optimal for both MLK and LCZ, yielding RMSECV values of 0.321 and 0.467 respectively, as depicted in (Fig. 5).

#### GA-PLS model

While PLS demonstrated excellent analytical capabilities, model optimization was pursued using GA to further enhance predictive performance [15]. GA works by iteratively selecting subsets of variables that maximize a desired output—in this case cross-validated model accuracy on the calibration set. Several key parameters were systematically adjusted over multiple GA runs to enable effective navigation of the complex high-dimensional search space. Population size, mutation rate, convergence criteria and wavelength subset sizes were rigorously tuned based on RMSECV patterns. As shown in (Table 2), a population of 40 chromosomes with 65 generations using 80% convergence threshold achieved high-resolution wavelength selection. Through this optimization, the absorption matrix was refined by discarding uninformative spectral regions containing minimal analyte signals. This reduced the matrix size by 63% and 51% for MLK and LCZ respectively, retaining only the most relevant variables. This GA-filtered spectrum was then utilized to reconstruct enhanced GA-PLS models using the same PLS routine. Cross-validation assessed the ideal number of LVs. for the reconstructed models, determining 2 LVs yielded optimal RMSECV of 0.221 and 0.307 for MLK and LCZ respectively, as shown in (Fig. 5). The Chemometric methods recommended in this study were applied to the calibration data using the optimal parameters. The concentrations of each component in the calibration set, consisting of 25 mixtures, were computed and presented in (Table 3). The predicted and known concentrations of each component were found to be linearly related. The GA-PLS model demonstrated superior performance by tailoring the spectral data matrix to retain only significant concentration-predictive signals. Sensitivity also improved since minimal wavelengths enabled reduced noise interference.

**Table 2:** Optimized parameters of GA selected as variable selection procedure to enhance the models' predictability

Parameters	Optimum values	
	MLK	LCZ
Population size	40	36
Maximum generations	65	52
Mutation rate	0.005	0.005
% wavelength used at the initiation	15	15
The number of variables in a window (window width)	2	2
Percent of the population (% of convergence)	80	80
Cross-type	Double	Double
Maximum number of latent variables	3	3
Cross-validation	Random	Random
Number of subsets to divide data into for cross-validation	5	5
Number of iterations for cross-validation at each generation	2	2

**Table 3:** Determination of MLK and LCZ in the calibration and validation set of the suggested methods

		CLS		PCR		PLS		GA-PLS	
		MLK	LCZ	MLK	LCZ	MLK	LCZ	MLK	LCZ
Calibration set	Mean	99.27	99.76	100.07	99.43	99.84	99.63	99.76	100.04
	SD	1.6282	1.506	1.606	1.595	1.3436	1.5149	1.0846	1.4768
	%RSD	1.6402	1.5096	1.6049	1.6041	1.3458	1.5205	1.0872	1.4762
	RMSEC^(a)^	0.1249	0.2592	0.2191	0.2023	0.2705	0.2904	0.0943	0.1926
Validation set	Mean	99.60	99.61	100.15	99.56	99.70	99.53	99.72	99.79
	SD	1.1662	0.9201	1.2701	0.6832	1.3381	0.9161	1.0342	0.5252
	%RSD	1.1709	0.9237	1.2682	0.6862	1.3421	0.9204	1.0371	0.5263
	RMSEP^(b)^	0.2904	0.2583	0.2843	0.2342	0.2742	0.3442	0.1872	0.1779

^a^Root Mean Square Error of calibration

^b^Root Mean Square Error of predication

#### Comprehensive validation of chemometric models

We implemented a systematic validation protocol spanning key analytical performance dimensions to rigorously assess the optimized models’ suitability for pharmaceutical quantification. We evaluated calibration set model fitting via the determination coefficient R^2^ and predictive ability using Q^2^. As shown in (Additional file 1: Table S1), R^2^ and Q^2^ exceeded 0.9 across all models, demonstrating excellent explanatory and predictive power on calibration samples. Additionally, the small gap between R^2^ and Q^2^ indicates good generalizability and minimal overfitting for the developed models. Additionally, The SEC values below 0.3 observed across models indicate excellent fitting and minimal deviations between predicted and reference concentrations on the calibration samples. Regarding external validation set performance, as shown in (Table 3 and Additional file 1: Fig. S1), excellent recoveries from 98 to 102% were obtained across all components and models, evidencing the excellent resolution capacity and minimal bias as well as the low RMSEP values below 0.3 affirm the high accuracy of concentration predictions on the independent LHS samples. Additionally, the RRMSEP values below 1.3% relative to the mean analyte concentrations attest to excellent predictive precision. The minute positive BCMSEP values approaching zero further validate the high predictive accuracy and negligible systematic quantification errors. Sensitivity was verified through low LOD and LOQ, meeting pharmaceutical analysis criteria. Method accuracy was systematically confirmed across the linear range through recovery testing at three concentrations in triplicate, with percentage recoveries from 98–102%. The repeatability and intermediate precision analyses demonstrated %RSD values below 2%, highlighting stable quantification despite short-term and long-term measurement timescale variations. Finally, unchanged performance was attained under intentionally altered conditions, establishing operational robustness, as shown in (Additional file 1: Table S1). Among the four developed chemometrics models, the GA-PLS approach consistently achieved the best results across all validation parameters, quantitatively confirming its optimal predictive capabilities for pharmaceutical analysis. Specifically, the GA-PLS model attained the highest R^2^ and Q^2^ (R^2^ = 0.9932 and 0.9901; Q^2^ = 0.9687 and 0.9542 for MLK and LCZ respectively), signifying its unrivaled data fitting and predictive accuracy. Additionally, it demonstrated the lowest RMSEP of just 0.1872 and 0.1779 for the two drugs, along with the lowest relative RRMSEP of 0.7516% and 0.6585%, highlighting top-tier generalization ability to new samples. The GA-PLS model also showed the best repeatability and intermediate precision with %RSDs not exceeding 0.9%, compared to under 2% for the other models. This underscores its superior analytical precision. Furthermore, it required the lowest detection limits (LOD = 0.0813 and 0.2273 μg/mL) to reliably quantify the pharmaceuticals, exhibiting the best sensitivity. Finally, it attained slightly higher recovery percentages (99.72–100.86%) and lower standard deviations (< 0.6%) during accuracy assessment, surpassing all models in analytical accuracy.

#### Statistical analysis

There is no notable distinction in accuracy between the different models, as indicated by the one-way ANOVA analysis of the validation data. The computed f values are lower than the critical f value, and the p values are greater than 0.05.; this suggests that the proposed models did not differ significantly from each other in terms of accuracy, as shown in (Additional file 1: Table S2). Additionally, the proposed chemometric methods were comparable to the reported method [21]. for determining MLK and LCZ, as shown in (Additional file 1: Table S2).

#### Assay of pharmaceuticals

The suggested approach was suitable for examining Montair-LC® tablets without any disruption from the additives. To validate the proposed method, a standard addition technique was employed, and the results are outlined in (Table 4).

**Table 4:** Determination of MLK and LCZ by the suggested chemometric methods and application of standard addition technique

Preparation		%Recovery ± %RSD ^(a)^			
		CLS	PCR	PLS	GA-PLS
MLK	Application	99.59 ± 0.907	99.60 ± 0.906	100.07 ± 1.292	99.53 ± 0.697
	Standard addition	98.21 ± 0.439	98.19 ± 0.439	100.24 ± 0.534	98.72 ± 0.854
LCZ	Application	99.62 ± 0.864	99.62 ± 0.864	100.1 ± 1.001	99.77 ± 0.754
	Standard addition	99.60 ± 0.662	99.59 ± 0.662	100.30 ± 0.665	99.26 ± 0.838

^a^Average of three determinations

#### Comparative study

Our systematic investigation elucidated the capabilities and limitations of the CLS, PCR, PLS and GA-PLS approaches for green pharmaceutical analysis. While all models demonstrated good predictive performance, notable divergence was observed in critical aspects like accuracy, sensitivity, precision, and applicability. The CLS technique showed satisfactory predictions but was constrained by its need for highly pure reference spectra and inability to resolve unknown interferences—impeding widespread quality control adoption. In contrast, PCR and PLS proved more flexible by deriving latent variables through multivariate decomposition of spectral data—bypassing exhaustive calibration component knowledge requirements. However, PCR retained excessive uninformative spectral variables compared to PLS which selectively captures concentration-correlated information, enhancing predictive robustness. Significantly, integrating GA variable selection with PLS unlocked decisive performance gains over raw data models. By intelligently filtering out spectral regions with negligible analyte signals, GA-PLS improved resolution and information extraction using fewer, optimized variables—boosting accuracy, precision, and sensitivity and enabling reliable applicability despite unknown matrix components. Quantitatively, this was evidenced in the GA-PLS model attaining the best RMSEC, SD, RMSCP, R% values, and other validation parameters, as shown in (Additional file 1: Table S1), marginally but consistently outperforming its PCA and PLS counterparts. In totality, while all models showed promise, the GA-PLS methodology systematically emerged as the definitive optimized approach, leveraging the strengths of multivariate data analysis and variable selection to deliver a practical, eco-friendly solution for accurate, green pharmaceutical quality control.

### Greenness, blueness, and whiteness tools

A multifaceted approach was undertaken to evaluate sustainability across critical dimensions like greenness, waste minimization, safety, analytical performance, and cost-effectiveness. Since no single tool provides comprehensive coverage, we applied a combined toolkit methodology for a more holistic assessment.

#### NEMI tool

The NEMI analysis offered an initial screening of greenness deficiencies through a visual format separated into four quadrants [9]. These four quadrants are 1-PBT (persistent, bioaccumulative, and toxic), 2-Hazardous, 3-Corrosive, and 4-Waste. The green quadrant indicates that 1- the reagents employed are not classified as PBT by the Environment Protection Agency's Toxic Release Inventory (EPA-TRI), 2- the chemicals utilized are non-hazardous and therefore not registered on the TRI list, 3- the medium pH falls between 2 and 12 and 4- less than 50g of waste is produced (Additional file 1: Fig. S1)*.* NEMI pictograms were created for the proposed method (Table 5). First sight at the pictograms showed that the proposed method was the greenest, meeting all NEMI criteria with four green quadrants.

**Table 5:**
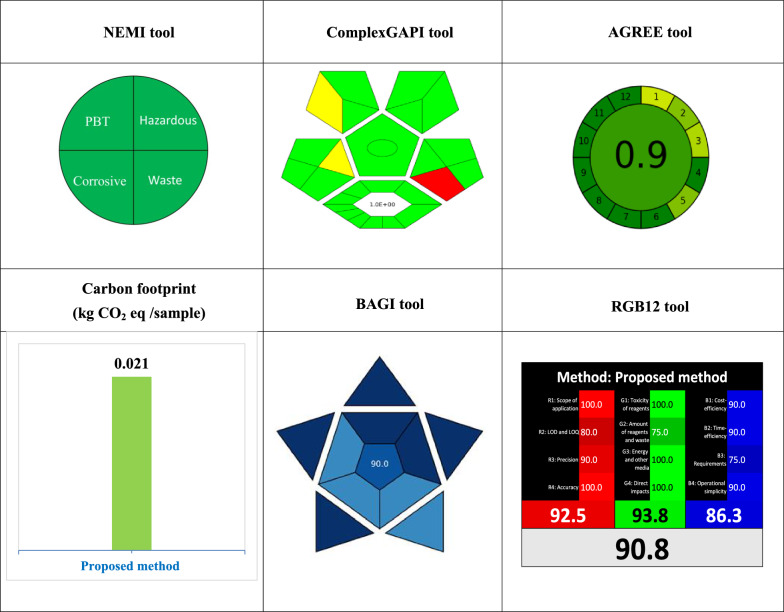
Greenness blueness and whiteness assessment of the proposed approach according to NEMI, ComplexGAPI, AGREE, carbon footprint, BAGI, and RGB12 tools

#### Complex GAPI tool

While NEMI enables initial greenness screening, the ComplexGAPI tool allows more comprehensive semi-quantitative evaluation [9]. It improves on the original GAPI metric by adding a hexagonal area representing the stages and procedures before the analytical methodology (Additional file 1: Fig. S2). This innovative tool encompasses all aspects of an analytical procedure, from collecting and transporting samples to their preservation, storage, preparation, and ultimate analysis [32]. ComplexGAPI also provides user-friendly software to construct visual pictograms. For this study, the proposed method demonstrated substantial advantages with a lower E-factor of 1 and predominance of favorable green icons signifying minimal waste generation and positive sustainability impact, as shown in (Table 2). However, ComplexGAPI focuses chiefly on environmental considerations. To enable a fuller assessment encompassing multi-dimensional sustainability metrics like waste minimization, energy efficiency, and renewable material usage, it proves advantageous to integrate ComplexGAPI with complementary quantitative tools.

#### AGREE tool

The AGREE metric provides a valuable quantitative approach for evaluating greenness by encompassing all 12 principles of GAC [9]. This enables thorough assessment grounded in widely accepted GAC criteria. A key advantage of AGREE is flexibility through customizable weighting of these diverse parameters. The user-friendly software converts the 12 inputs into a single score from 0 to 1, visualized on a colored pictogram for rapid interpretation. Dark green indicates excellent greenness while dark red signifies major deficiencies. For this study, the proposed method achieved a high AGREE score of 0.90 (Table 2), affirming remarkable efficacy in advancing sustainability aims. However, AGREE focuses solely on environmental factors. To enable a holistic sustainability evaluation, it proves beneficial to integrate AGREE with tools assessing other critical aspects like safety, analytical performance, practicality, and cost-effectiveness.

#### Carbon footprint analysis

Unlike other greenness assessments, carbon footprint analysis enables quantitative comparison of analytical methods' environmental impacts in terms of greenhouse gas emissions, reported as kilograms of CO_2_ equivalent [9]. By capturing critical aspects like electricity usage, reagent transportation, and waste generation in a composite measure, carbon footprint effectively complements tools like NEMI, ComplexGAPI, and AGREE that lack quantitative emissions estimation. We calculated the carbon footprint using the standardized equation below [9]:

$$\mathrm{Carbon\,footprint }\left(\mathrm{kg CO}2\mathrm{ eq}\right)=\sum Instrument\,Power \left(kW\right) . Analysis\,time \left(h\right) . Emission\,factor (kg CO2/kWh)$$

The proposed method demonstrated a markedly lower carbon footprint of just 0.021 kg CO_2_ eq per sample, as shown in Table 2. The lower carbon footprint of our technique is attributed to the reduced electricity consumption enabled by shorter analysis times and the absence of a derivatization step. Moreover, replacing hazardous solvents like chloroform and methylene chloride with water markedly decreased transport-related emissions, confirming its favorable environmental profile.

#### BAGI tool

Unlike predominantly greenness-focused tools, the recently introduced BAGI metric delivers a quantitative evaluation of an analytical method's “blueness”—defined as its real-world fitness for purpose based on critical practical criteria [33]. BAGI enables a comprehensive assessment of an analytical method's blueness or applicability by considering ten key parameters: analysis type, number of analytes, instrumentation, sample throughput, sample preparation needs, samples analyzed per hour, reagents/materials required, preconcentration needs, degree of automation, and sample amount needed. Each of these ten factors is scored on a scale of 1 (worst) to 10 (best). The composite BAGI score computes the geometric mean of the ten individual criteria scores. A higher BAGI score indicates a more applicable, functional, and fit-for-purpose analytical method. Our method obtained a high BAGI score of 90, indicating excellent blueness. The BAGI assessment validates that our technique offers significant advantages in terms of time and cost savings, hazard minimization, and overall functionality, as depicted in (Table 2). However, while BAGI evaluates critical real-world applicability, it does not provide a complete, holistic quantification of sustainability. To achieve a more comprehensive assessment encompassing greenness, analytical merit, and practicality, we additionally applied the RGB12 algorithm.

#### RGB12 tool

The RGB12 tool was introduced in June 2021 by Paweł-Nowak and his team [34]. It is a quantitative assessment tool that is easy to use for evaluating whiteness. This tool determines the degree of sustainability concerning whiteness assessment and assesses methods based on the 12 WAC considerations. [9]. The RGB12 algorithm comprises twelve distinct algorithms organized into three groups: red, green, and blue, where each group consists of four algorithms. The green subgroup (G1-G4) deals with important GAC parameters like toxicity, amount of waste and reagent, energy conservation, and influences on humans, animals, and genetic alterations. The red subgroup (R1-R4) focuses on validation parameters such as application scope, accuracy, LOD, precision, and LOQ. The blue subgroup (B1-B4) concerns practical and economic necessities, cost-effectiveness, and time efficiency. The RGB12 algorithm adds the method's scores in each of the three-color areas to determine the final "whiteness" value, which shows how the method adheres to WAC concepts. The suggested method exhibits a remarkable whiteness score of 90.8, as displayed in (Table 2), this demonstrates that the method has numerous benefits with regard to environmental friendliness, sustainability, economic viability, practicality, and analytical efficiency. Using RGB12 with other metrics provided a comprehensive, robust sustainability evaluation and avoided single technique limitations. This systems-thinking attitude using various complementary tools represents a best practice for detailed, unbiased analytical method sustainability assessment.

## Recommendations for future work and limitations

While the developed methods demonstrate strong performance and sustainability advantages, further enhancement is possible. Additional analytes and matrices could be explored to expand applicability. To address the industrial application capacity, based on Richard G. Brereton's work on multilevel multifactor designs, a key recommendation is to investigate expanding the total number of samples that can be robustly measured simultaneously in a single mixture to up to 24 analytes using these chemometric techniques. This would significantly enhance throughput and efficiency compared to conventional methods analyzing one sample at a time.

Regarding limitations, The UV–Vis technique provides limited structural information compared to techniques like NMR and mass spectrometry, restricting its applicability mainly to quantitative analysis rather than the structural elucidation of pharmaceuticals. Spectral overlaps between analytes can still occur, requiring mathematical resolution via chemometric tools. In some cases, partial separation using selective solvents or pH adjustment may be beneficial before spectral acquisition. Sample matrix interferences pose challenges for accurate quantification in complex formulations, necessitating appropriate sample preparation or pre-treatment strategies tailored to the matrix.

Despite the limitations outlined, the simplicity, affordability, speed, and eco-friendliness position our chemometric strategy as a promising solution, especially for laboratories with moderate instrumental facilities. Addressing the challenges highlighted here through future research will expand the scope and refine the utility of sustainable chemometric models in pharmaceutical analysis.

## Conclusion

This work has successfully developed and validated the first chemometric models for the simultaneous quantification of two structurally dissimilar anti-COVID drugs, MLK and LCZ which align with sustainable development objectives. The proposed CLS, PCR, PLS and GA-PLS models demonstrated excellent predictive capabilities, accuracy, precision, sensitivity, and robustness for pharmaceutical analysis. The optimized GA-PLS model demonstrated superior performance with excellent accuracy, precision, and minimal environmental impact. A key highlight was the implementation of a cutting-edge statistical design technique called LHS to construct an optimal validation set. LHS provided a rigorous, unbiased assessment of the models' ability to generalize across the full concentration range, overcoming limitations in chemometrics where studies predominantly use random data splitting. By enhancing predictive reliability with fewer validation samples, this aligns with green analytical principles of resource efficiency. Furthermore, this research spearheaded comprehensive quantitative greenness, blueness, and whiteness evaluations using state-of-the-art tools like NEMI, ComplexGAPI, AGREE, BAGI, and RGB12. Favorable results on critical parameters related to eco-friendliness, analytical performance, real-world applicability, affordability, and sustainability were achieved. Overall, the developed UV–vis chemometrics approach has shown immense promise as a rapid, inexpensive and sustainable quality control workflow amenable to widespread pharmaceutical implementation, even in laboratories with basic facilities. It can serve as a viable green alternative to costly chromatographic techniques. Additionally, this method has demonstrated significant advantages regarding time and cost savings, hazard minimization, analytical efficiency and practical functionality. Hence, it holds substantial utility for the regulated routine analysis of MLK and LCZ across quality control and research settings. By embracing greenness, blueness and whiteness perspectives, this work puts forward an eco-friendly, fit-for-purpose and value-centric direction for analytical progression that meaningfully furthers sustainable development aims.

### Supplementary Information

**Additional file 1: Fig. S1.** Typical NEMI pictograms. **Fig. S2.** The ComplexGAPI pictogram, with the original GAPI pictogram greyed out in the background, and particular fields of the added hexagonal glyph grouped and colour-coded for clarity. **Table S1. **Validation sheet and regression parameters of MLK, and LCZ by the proposed methods. **Table S2. **One-way ANOVA statistical analysis of the results obtained by applying the proposed Chemometric methods for the determination of MLK, and LCZ in pharmaceutical preparation by the proposed methods and the reported method within a 95% confidence limit.

## Data Availability

Data was collected using a spectrophotometer and software. The corresponding author will provide the datasets created and/or analyzed during the current study upon reasonable request.
