# Supplementary material for: Four chemometric models enhanced by Latin hypercube sampling design for quantification of anti-COVID drugs: sustainability profiling through multiple greenness, carbon footprint, blueness, and whiteness metrics
Source: BMC Chem. 2024 Mar 18;18(1):54. doi: 10.1186/s13065-024-01158-7 (PMC10949693; doi:10.1186/s13065-024-01158-7)
Supplement: Supplementary file 1 — Additional file 1: Fig. S1. Typical NEMI pictograms. Fig. S2. The ComplexGAPI pictogram, with the original GAPI pictogram greyed out in the background, and particular fields of the added hexagonal glyph grouped and colour-coded for clarity. Table S1. Validation sheet and regression parameters of MLK, and LCZ by the proposed methods. Table S2. One-way ANOVA statistical analysis of the results obtained by applying the proposed Chemometric methods for the determination of MLK, and LCZ in pharmaceutical preparation by the proposed methods and the reported method within a 95% confidence limit. [file 13065_2024_1158_MOESM1_ESM.docx]

**Additional file 1: Figures**


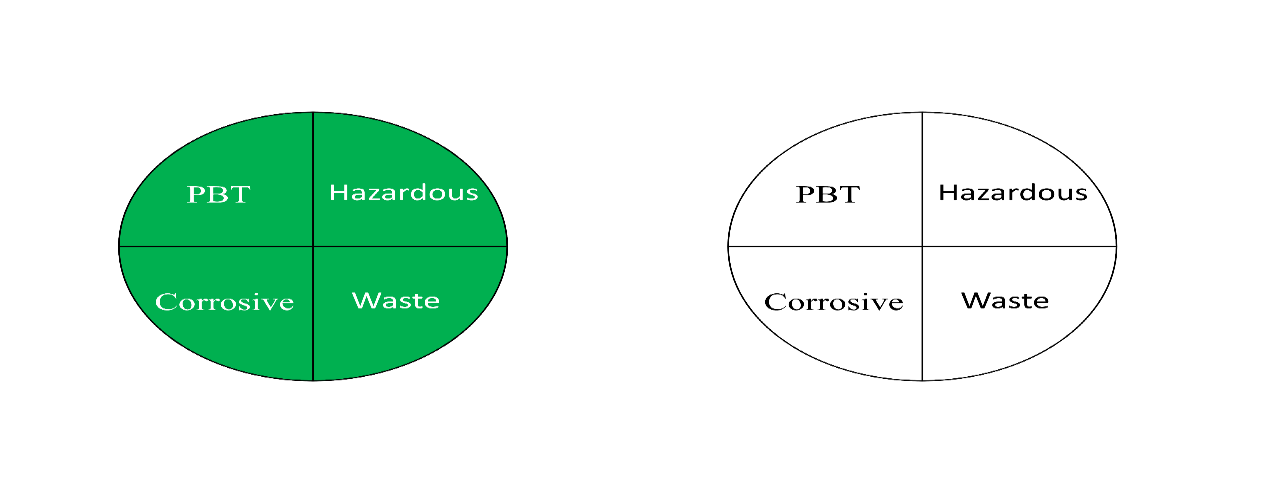


**Fig. S1.** Typical NEMI pictograms


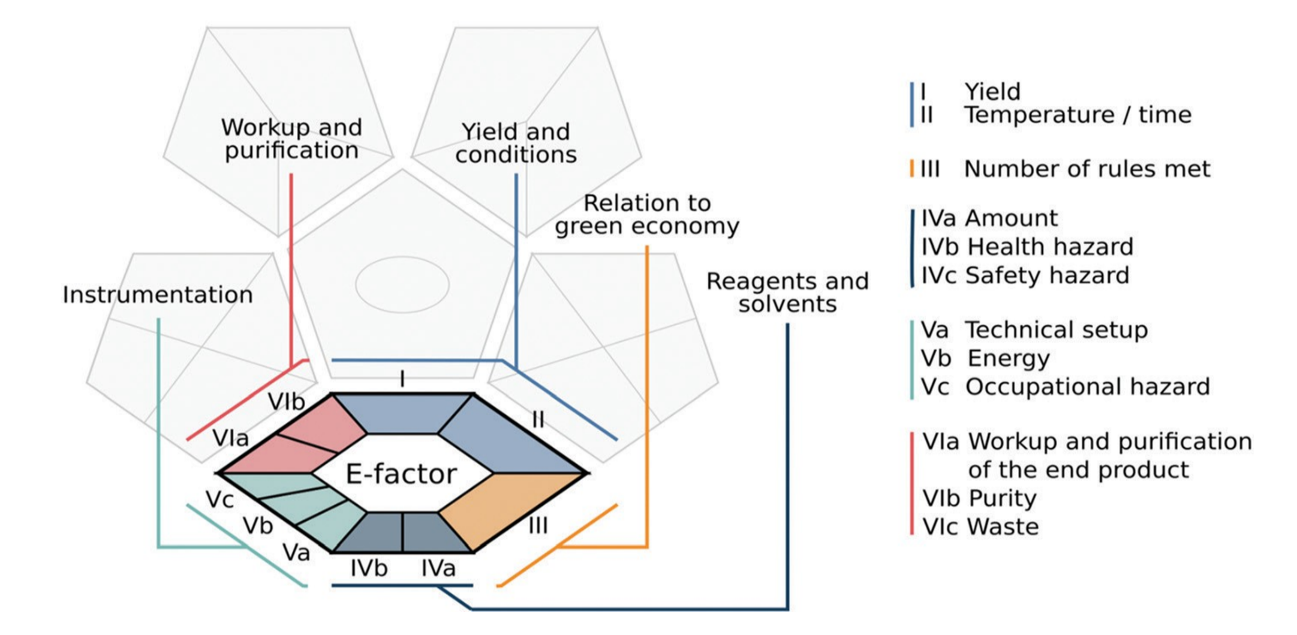


**Fig. S2.** The ComplexGAPI pictogram, with the original GAPI pictogram greyed out in the background, and particular fields of the added hexagonal glyph grouped and colour-coded for clarity.

**Tables**

**Table S1:** Validation sheet and regression parameters of MLK, and LCZ by the proposed methods.

| **parameters** | **CLS** | | **PCR** | |
| --- | --- | --- | --- | --- |
|  | **MLK** | **LCZ** | **MLK** | **LCZ** |
| **Range (μg/mL)** | 10–30 | 10–30 | 10–30 | 10–30 |
| **Slope ^a^** | 0.9813 | 0.9801 | 0.9821 | 0.9818 |
| **Intercept ^a^** | -0.0094 | -0.0218 | -0.0123 | -0.0239 |
| **R^2^** | 0.9841 | 0.9654 | 0.9891 | 0.9679 |
| **Q^2^** | 0.9012 | 0.8956 | 0.9543 | 0.9321 |
| **LOD (μg/mL) ^b^** | 0.0970 | 0.3597 | 0.0931 | 0.34496 |
| **LOQ (μg/mL) ^b^** | 0.2940 | 1.0899 | 0.2821 | 1.0453 |
| **RRMSEP ^c^** | 0.8310 | 0.9859 | 1.0760 | 1.2985 |
| **BCMSEP ^d^** | -0.0069 | 0.0392 | -0.0176 | 0.0686 |
| **SEC ^e^** | 0.2224 | 0.0647 | 0.2763 | 0.0695 |
| **Accuracy ^f^ (M.R % ± S.D)** | 99.28 ± 1.014 | 101.44 ± 0.894 | 99.49 ± 1.002 | 101.26 ± 0.626 |
| **Repeatability ^f^ (%RSD )** | 0.8644 | 0.5409 | 0.8075 | 0.5027 |
| **Intermediate precision ^f^ (%RSD )** | 0.9251 | 0.9907 | 0.9025 | 0.8828 |
| **Robustness ^f^ (%RSD )** | 1.4092 | 0.9662 | 1.1916 | 0.8058 |
|  | **PLS** | | **GA-PLS** | |
| **Parameters** |  |  |  |  |
|  | **MLK** | **LCZ** | **MLK** | **LCZ** |
| **Range (μg/mL)** | 10–30 | 10–30 | 10–30 | 10–30 |
| **Slope ^a^** | 0.9811 | 0.9808 | 0.9794 | 0.9817 |
| **Intercept ^a^** | -0.0093 | -0.0221 | 0.0046 | -0.0493 |
| **R^2^** | 0.9908 | 0.9823 | 0.9932 | 0.9901 |
| **Q^2^** | 0.9617 | 0.9479 | 0.9687 | 0.9542 |
| **LOD (μg/mL) ^b^** | 0.0970 | 0.3596 | 0.0813 | 0.2273 |
| **LOQ (μg/mL) ^b^** | 0.2940 | 1.0887 | 0.2459 | 0.6889 |
| **RRMSEP ^c^** | 0.8310 | 0.9819 | 0.7516 | 0.6585 |
| **BCMSEP ^d^** | -0.0068 | 0.0392 | -0.0029 | 0.0176 |
| **SEC ^e^** | 0.2224 | 0.0646 | 0.1548 | 0.0568 |
| **Accuracy ^f^ (M.R % ± S.D)** | 100.05 ± 0.599 | 100.96 ± 0.641 | 99.86 ± 0.429 | 100.86 ± 0.523 |
| **Repeatability ^f^ (%RSD )** | 0.8055 | 0.5007 | 0.7869 | 0.4860 |
| **Intermediate precision ^f^ (%RSD )** | 0.8986 | 0.9006 | 0.8810 | 0.8839 |
| **Robustness ^f^ (%RSD )** | 1.1731 | 0.8349 | 1.0887 | 0.7947 |

^a^ Data of the straight line plotted between predicted concentrations versus actual concentrations of the calibration set.

^b^ The LOD and LOQ calculations are based on the net analyte signals.

^c^ Relative root mean square error of prediction.

^d^ Bias corrected mean square error of prediction.

^e^ standard error of calibration

^f^ Average of three determinations.

**Table S2:** One-way ANOVA statistical analysis of the results obtained by applying the proposed Chemometric methods for the determination of MLK, and LCZ in pharmaceutical preparation by the proposed methods and the reported method within a 95% confidence limit.

| **Component** |  | **Sum of squares** | **df** | **Mean square** | **F** | **P value** |
| --- | --- | --- | --- | --- | --- | --- |
| **MLK** | **Between Groups** | 5.275 | 4.000 | 1.319 | 0.838 (2.866)^a^ | 0.517 |
|  | **Within Groups** | 31.466 | 20.000 | 1.573 |  |  |
|  | **Total** | 36.741 | 24.000 |  |  |  |
|  |  |  |  |  |  |  |
| **LCZ** | **Between Groups** | 9.341 | 4.000 | 2.335 | 2.100 (2.866)^a^ | 0.119 |
|  | **Within Groups** | 22.240 | 20.000 | 1.112 |  |  |
|  | **Total** | 31.580 | 24.000 |  |  |  |

^a^ Figures in parentheses represent the corresponding critical value of F at P < 0.05.


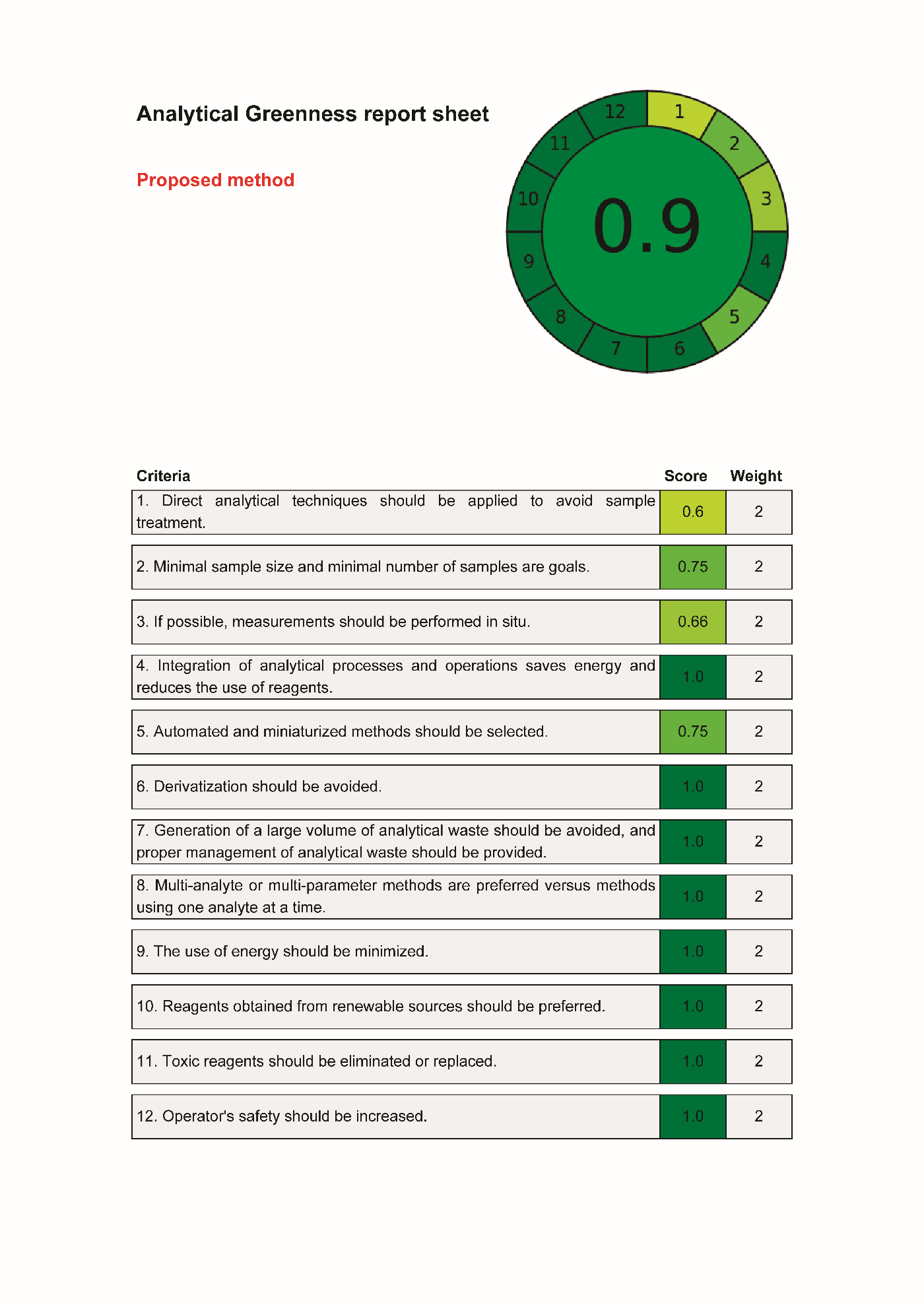
**Analytical Greenness report sheets for the suggested method by the AGREE tool**
